# Supplementary material for: Molecular and functional analysis of calcium binding by a cancer-linked calreticulin mutant
Source: eLife. 2026 Oct 29;15:RP109647. doi: 10.7554/eLife.109647 (PMC13719167; doi:10.7554/eLife.109647)
Supplement: Supplementary file 2 [file elife-109647-supp1.docx]

**Molecular and Functional Analysis of Calcium Binding by a Myeloproliferative Neoplasm-linked Calreticulin Mutant**

**Supplementary File**

This document contains supplementary tables

**Table A:** List of primers used in this study for cloning of the specified DNA constructs or site-directed mutagenesis as indicated

| **Name of the construct** | **Primers** | **Purpose** | **References** |
| --- | --- | --- | --- |
| pGB1-*CALR_(1-351)_* | *Forward*: 5’-CAGGACGAGGAGCAGAGGTAGAGGAGGATGATGAGGACA-3’ | Generate CRT (1-351) truncation mutant by site-directed mutagenesis | This study |
|  | *Reverse*: 5’-TGTCCTCATCATCCTCCTCTACCTCTGCTCCTCGTCCTG-3’ |  |  |
| pGB1*-CALR_(1-339)_* | *Forward*: 5’-TAACAAAGGCAGCAGAGAAATAAATGAAGGACAAACAGGAC-3’ | Generate CRT (1-339) truncation mutant by site-directed mutagenesis | This study |
|  | *Reverse*: 5'-GTCCTGTTTGTCCTTCATTTATTTCTCTGCTGCCTTTGTTA-3' |  |  |
| pLB-*CALR* sgRNA | *Forward* (**BsmBI**): 5’-CACCGGGCCACAGATGTCGGGACCT-3' | Cloning of *CALR*-sgRNA in pLentiCRISPRv2-BLAST (pLB) vector; the underlined bases correspond to the sgRNA sequence | 10.1074/jbc.RA120.014372 |
|  | *Reverse* (**BsmBI**): 5-AAACAGGTCCCGACATCTGTGGCCC-3’ |  |  |
| pSIP Vector minus *ZsGreen* | *Forward* (**PmeI**): 5’-AATTCccg**GTTTAAAC**gccG-3' | Replacement of the *ZsGreen* fragment of the pSIP-*ZsGreen* vector with the PmeI sequence | This study |
|  | *Reverse* (**PmeI**): 5’-Gggc**CAAATTTG**cggCCTAG-3' |  |  |
| pSIP-*CALR_WT_* | *Forward* (**EcoRI**): 5’-CGAA**GAATTC**GCCGCCACCATGCTGCTATCCG-3' | Cloning of *CALR_WT_* cDNA in pSIP vector; the underlined sequence in forward primer is the Kozak sequence | This study |
|  | *Reverse* (**NotI**): 5’-GCATTATT**GCGGCCGC**CTACAGCTCGTCCTTGGC-3' |  |  |
| pSIP-*CALR_Del52_* | *Forward* (**EcoRI**): 5’-CGAA**GAATTC**GCCGCCACCATGCTGCTATCCG-3' | Cloning of *CALR_Del52_* cDNA in pSIP vector; the underlined sequence in the forward primer is the Kozak sequence | This study |
|  | *Reverse* (**NotI**): 5’-GCATTATT**GCGGCCGC**TCAGGCCTCAGTCCAGCC-3' |  |  |
| pSIP-*CALR_Del52+KDEL_* | *Forward* (**EcoRI**): 5’-CGAA**GAATTC**GCCGCCACCATGCTGCTATCCG-3' | Cloning of *CALR_Del52_*_+KDEL_ cDNA in pSIP vector; the underlined sequence in forward primer is the Kozak sequence | This study |
|  | *Reverse* (**NotI**): 5’-GA**GCGGCCGC**TCATAATTCATCTTTGGCCTCAGTC-3' |  |  |
| sgRNA-resistant pSIP-*CALR_WT_* and pSIP-*CALR_Del52_* | C1QF (*Forward*): ATACAACATCATGTTTGGGCCTGATATCTGTGGCCCTGGCACC | Site-directed mutagenesis to make pSIP-*CALR_WT_* and pSIP-*CALR_Del52_* constructs sgRNA resistant | This study |
|  | C1QR (*Reverse*): GGTGCCAGGGCCACAGATATCAGGCCCAAACATGATGTTGTAT |  |  |
|  | C2QF (*Forward*): CAACATCATGTTTGGGCCTGATATATGCGGACCTGGCACCAAGA |  |  |
|  | C2QR (*Reverse*): TCTTGGTGCCAGGTCCGCATATATCAGGCCCAAACATGATGTTG |  |  |

**Table B**: List of primers used for amplification of target genes in the qPCR assays

| **Primers** | **Target gene** | **References** |
| --- | --- | --- |
| *Forward*: 5’-GATGAAGCTCTCCCTGGTGG-3’ | *HSPA5* | This study |
| *Reverse*: 5’-TAGGTGGTCCCCAAGTCGAT-3’ |  |  |
| *Forward*: 5’-GGAGAGTCGTGAAGCAGTTGAG-3’ | *HSP90B1* | This study |
| *Reverse*: 5'-CCACCAAAGCACACGGAGATTC-3' |  |  |
| *Forward*: 5’-GTCAGCCACTTGAAGAAGCAGG-3’ | *PDIA3* | This study |
| *Reverse*: 5'-TAGGAACTCGGAGTGAGCCTCA-3' |  |  |
| *Forward*: 5’-TCAGAAAGGCGAGTCTCCTGTG-3’ | *PDIA6* | This study |
| *Reverse*: 5'-CCTCTTGGCAATGTCCTCGTTG-3' |  |  |
