# Supplementary material for: Molecular and functional analysis of calcium binding by a cancer-linked calreticulin mutant
Source: eLife. 2026 Oct 29;15:RP109647. doi: 10.7554/eLife.109647 (PMC13719167; doi:10.7554/eLife.109647)
Supplement: Supplementary file 3 [file elife-109647-fig2-data2.zip › Figure 2B_Source data 2/Figure 2B_data.pdf]

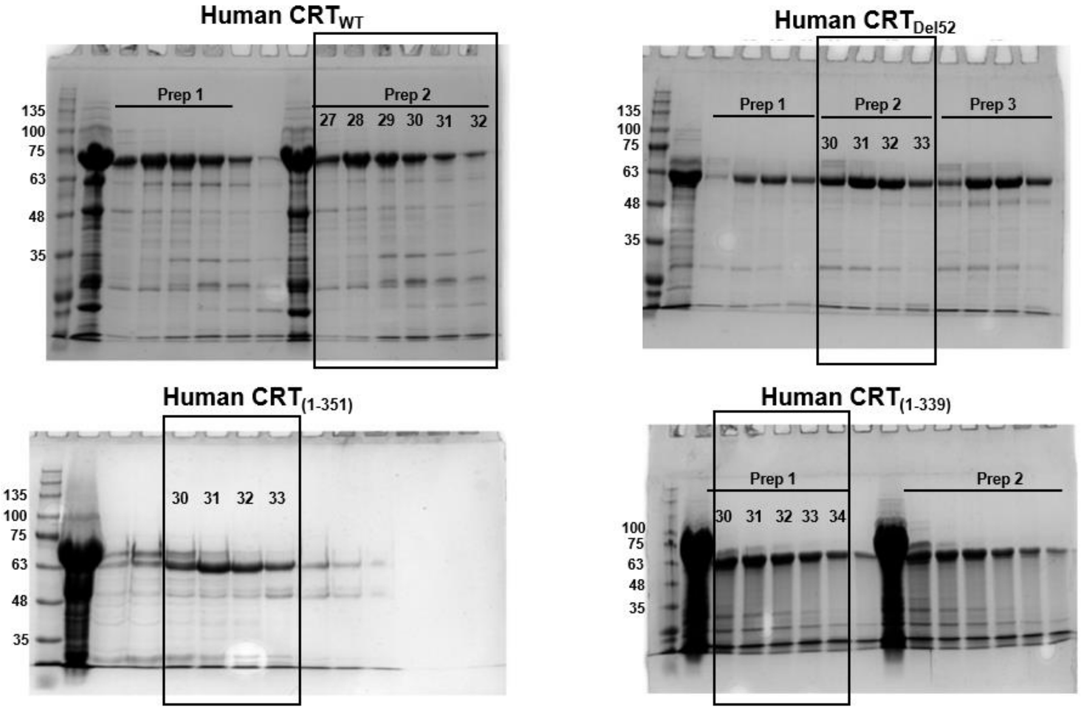
Human CRT_{WT}
Prep 1
Prep 2
27 28 29 30 31 32
135
100
75
63
48
35
Human CRT_{Del52}
Prep 1
Prep 2
Prep 3
30 31 32 33
135
100
75
63
48
35
Human $CRT_{(1-351)}$
30 31 32 33
135
100
75
63
48
35
Human $CRT_{(1-339)}$
Prep 1
Prep 2
30 31 32 33 34
100
75
63
48
35
