# Supplementary material for: Molecular and functional analysis of calcium binding by a cancer-linked calreticulin mutant
Source: eLife. 2026 Oct 29;15:RP109647. doi: 10.7554/eLife.109647 (PMC13719167; doi:10.7554/eLife.109647)
Supplement: Supplementary file 5 [file elife-109647-fig4-data1.zip › Figure 4A_Source Data-1/Figure 4A.pdf]

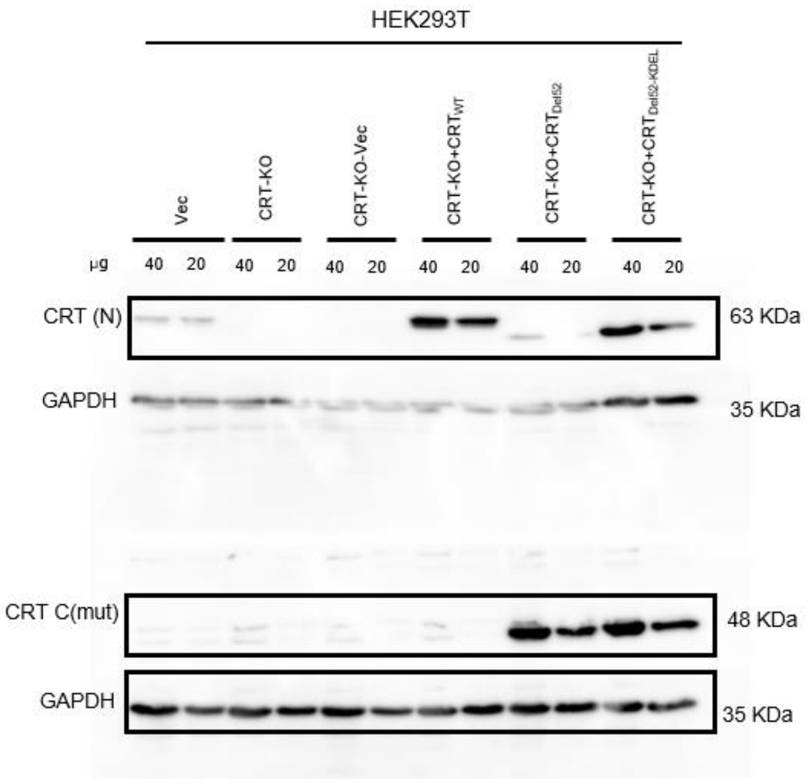
HEK293T
Vec
CRT-KO
CRT-KO-Vec
CRT-KO+CRT$_{WT}$
CRT-KO+CRT$_{Del52}$
CRT-KO+CRT$_{Del52-KDEL}$
μg
40 20 40 20 40 20 40 20 40 20 40 20
CRT (N)
63 KDa
GAPDH
35 KDa
CRT C(mut)
48 KDa
GAPDH
35 KDa
