# Supplementary material for: Molecular and functional analysis of calcium binding by a cancer-linked calreticulin mutant
Source: eLife. 2026 Oct 29;15:RP109647. doi: 10.7554/eLife.109647 (PMC13719167; doi:10.7554/eLife.109647)
Supplement: Supplementary file 7 [file elife-109647-fig6-data1.zip › Figure 6A_Source data 1/Figure 6A.pdf]

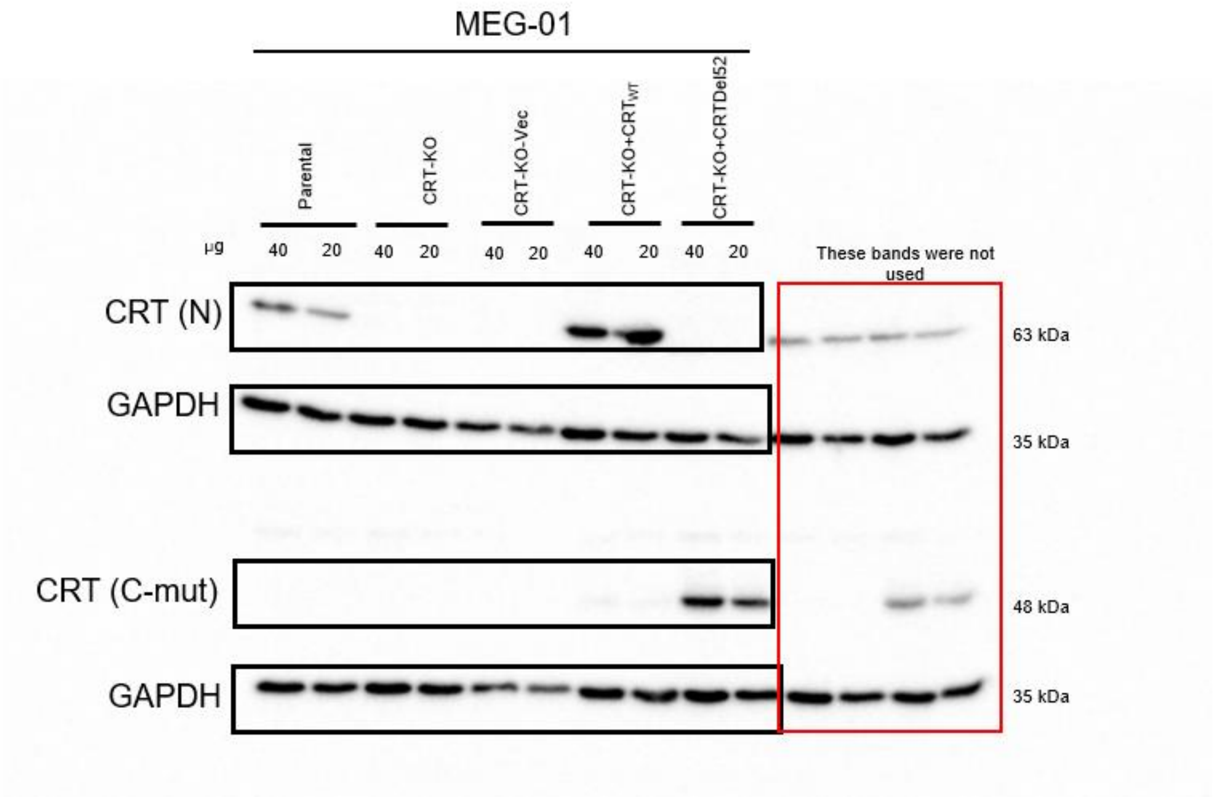
MEG-01
Parental
CRT-KO
CRT-KO-Vec
CRT-KO+CRTWT
CRT-KO+CRTDel52
µg
40 20 40 20 40 20 40 20 40 20
These bands were not used
CRT (N)
GAPDH
CRT (C-mut)
GAPDH
63 kDa
35 kDa
48 kDa
35 kDa
