# Supplementary material for: Molecular and functional analysis of calcium binding by a cancer-linked calreticulin mutant
Source: eLife. 2026 Oct 29;15:RP109647. doi: 10.7554/eLife.109647 (PMC13719167; doi:10.7554/eLife.109647)
Supplement: Supplementary file 11 [file elife-109647-fig6-figsupp1-data1.zip › Figure 6-fig supplement-source data 1/Figure 6_Figure supplement 1.pdf]

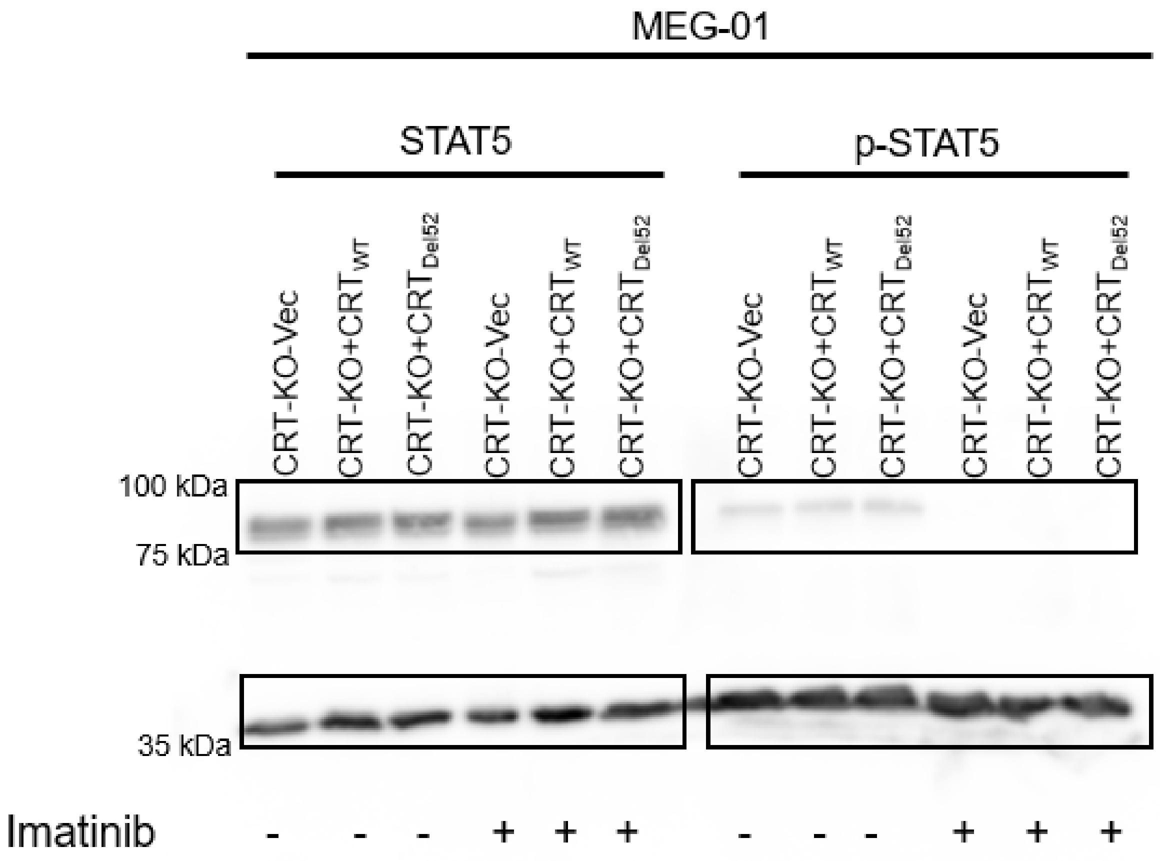

MEG-01
STAT5
p-STAT5
CRT-KO-Vec
CRT-KO+CRT$_{WT}$
CRT-KO+CRT$_{Del52}$
CRT-KO-Vec
CRT-KO+CRT$_{WT}$
CRT-KO+CRT$_{Del52}$
CRT-KO-Vec
CRT-KO+CRT$_{WT}$
CRT-KO+CRT$_{Del52}$
CRT-KO-Vec
CRT-KO+CRT$_{WT}$
CRT-KO+CRT$_{Del52}$
100 kDa
75 kDa
35 kDa
Imatinib
- - - + + + - - - + + +
