# Supplementary material for: Molecular and functional analysis of calcium binding by a cancer-linked calreticulin mutant
Source: eLife. 2026 Oct 29;15:RP109647. doi: 10.7554/eLife.109647 (PMC13719167; doi:10.7554/eLife.109647)

BiP

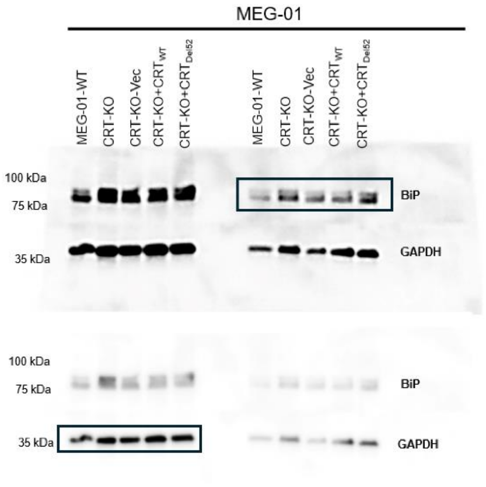

GRP94

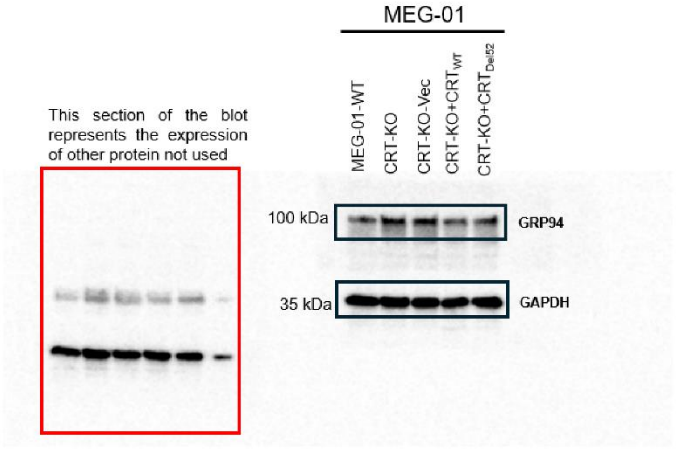

PDIA6

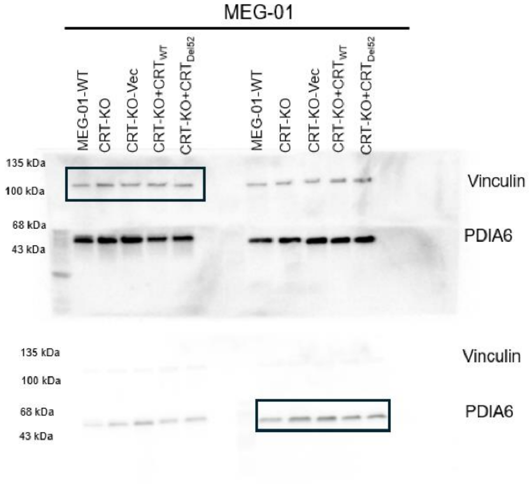

PDIA3

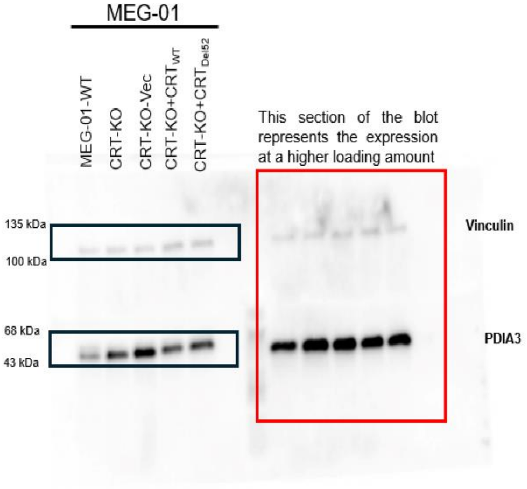

Supplement: Supplementary file 17 [file elife-109647-fig8-data3.zip › Figure 8D-G [right panels]-source data 1/Figure 8D-G[right panels].pdf]
