# Supplementary material for: Molecular and functional analysis of calcium binding by a cancer-linked calreticulin mutant
Source: eLife. 2026 Oct 29;15:RP109647. doi: 10.7554/eLife.109647 (PMC13719167; doi:10.7554/eLife.109647)
Supplement: Peer review history

# Editor report: eLife Assessment

**DOI:** 10.7554/eLife.109647.3.sa0

**Authors:** Michael L Dustin

**Affiliations:** University of Oxford United Kingdom

**Keywords:** Convincing, Important

This work makes an **important** contribution to understanding the role of calreticulin and the Del52 variant in calcium homeostasis in cells based on **convincing** evidence. There was some question as to whether the work in vitro fully recapitulated the physiological setting in which the Del52 variant has been reported to have defects in calcium homeostasis. Nonetheless, the studies are well performed using appropriate methods to address these questions and make a significant contribution to the understanding of calreticulin, and the Del52 variant, function in health and disease.

# Referee report: Reviewer #1 (Public review)

**DOI:** 10.7554/eLife.109647.3.sa1

The authors attempted to compare calcium binding properties of wildtype calreticulin with calreticulin deletion mutant (CRTDel52) associated with myeloproliferative neoplasms.

The researchers conducted their study using advanced techniques They found almost no difference in calcium binding between the two proteins and observed no impact on calcium signaling, specifically store-operated calcium entry (SOCE). The study also noted an increase in ER luminal calcium-binding chaperone proteins. Surprisingly, the authors selected flow cytometry as a technique for measurements of ER luminal calcium. Considering limitations of this approach it would be better to use alternative approaches. This is particularly important as previous reports, using cells from MPN patients, indicate reduced ER luminal calcium and effects on SOCE (Blood, 2020). This issue matters because earlier research with MPN patient cells reported reduced ER luminal calcium levels and altered SOCE (Blood, 2020). How do the authors explain the difference between their results and previous findings about lower ER luminal calcium and changed SOCE in MPN patient cells expressing CRTDel52? Other studies have found that unfolded protein responses are activated in MPN cells with CRTDel52 calreticulin (see Blood, 2021), and increased UPR could account for higher levels of some ER resident calcium-binding proteins observed here. Overall, it remains unclear how this work improves our understanding of MPN or clarifies calreticulin's role in MPN pathophysiology.

Comments on revised version.

The authors have addressed the points raised in the original review. However, given the absence of significant differences between the wild-type and mutant proteins, the relevance of this work to MPN pathology remains unclear. The novelty of the study is limited, as calcium has generally not been considered a significant factor in MPN pathology associated with mutant calreticulin.

# Referee report: Reviewer #2 (Public review)

**DOI:** 10.7554/eLife.109647.3.sa2

Summary:

Tagoe and colleagues present a thorough analysis of the calcium (Ca2+) binding capacity of calreticulin (CRT), an endoplasmic reticulum (ER) Ca2+-buffer protein, using a mutant version (CRT del52) found in myeloproliferative neoplasms (MPNs). The authors use purified human CRT protein variants, CRT-KO cell lines, and an MPN cell line to elucidate the differing Ca2+ dynamics, both on the level of the protein and on cell-wide Ca2+-governed processes. In sum, the authors provide new insights into CRT that can be applied to both normal and malignant cell biology.

First the authors purify CRT protein and perform isothermal titration calorimetry to quantify the Ca2+ binding capacity of CRT. They use full-length human CRT, CRT del52, and two truncations of CRT (1-339 and 1-351, the former of which should lead to the entire loss of low affinity Ca2+ binding). While CRT del52 has previously been shown to lead to a decrease in Ca2+ binding affinity in other models, the ITC data shows that this is retained in CRT del52.

Next, the authors utilize a CRT-KO cell line with subsequent addition of CRT protein variants to validate these findings with flow cytometric analysis. Cells were transfected with a ratiometric ER Ca2+ probe, and fluorescence indicates that CRT del52 is unable to restore basal ER Ca2+ levels to the same extent as CRT wild-type. To translate these findings to MPNs, the authors perform CRT-KO in a megakaryocytic cell line, where reconstitution with either CRT variant did not cause a difference in cytosolic calcium levels. The authors further test store-operated calcium entry (SOCE), an important process to maintaining ER Ca2+ levels, in these cells, and find that CRT-KO cells have lower SOCE activity, and that this can be slightly recovered with CRT addition.

Finally, the authors ask whether other effects of CRT-KO/reconstitution can affect cellular Ca2+ signaling pathway and levels. RNASeq analysis revealed showed that CRT-KO lead to an increase in various chaperone protein expressions, and that reconstitution with CRT del52 is unable to reduce expression to the same extent as reconstitution with CRT wildtype.

Comments on revised version.

The authors have sufficiently addressed my concerns from the first review.

# Author comment: Author response

**DOI:** 10.7554/eLife.109647.3.sa3

**Authors:** Ishmael Nii Ayibontey Tagoe, Amanpreet Kaur, Osbourne Quaye, Emmanuel Ayitey Tagoe, Nicole M Koropatkin, Leslie S Satin, Malini Raghavan

**Affiliations:** University of Michigan Ann Arbor United States; University of Michigan Ann Arbor United States; University of Ghana Legon Ghana; University of Ghana Legon Ghana; University of Michigan Medical School Ann Arbor United States; University of Michigan Ann Arbor United States; University of Michigan Ann Arbor United States

The following is the authors’ response to the original reviews.

**eLife Assessment**

This study investigates low-affinity Ca2+ binding by WT calreticulin and mutant calreticulin associated with type I myeloproliferative neoplasms, as well as the impact on Ca2+ fluxes in suspension cultures of megakaryocyte-like cells in vitro in response to ER Ca2+ ATPase inhibitors that deplete endoplasmic reticulum (ER) Ca2+ store and open plasma membrane Ca2+ channels through STIM1-Orai interactions. The results are important in that they show that Ca2+ binding by calreticulin and store-operated Ca2+ entry are not fundamentally impacted by the type I deletion mutation in calreticulin, which rules out a direct effect of the calreticulin mutation on its own low-affinity Ca2+ binding and any broad impact on ER Ca2+ regulation. The strength of the data and methods used ranges from solid to convincing, although the use of suspension-based flow cytometric assays to investigate ER Ca2+ levels and Ca2+ entry can be challenged. High-affinity Ca2+ binding sites could be further considered, and possible confounding effects of Abl kinase activity in the megakaryocyte-like cell lines could be offset.

The authors thank the editors and the reviewers for the summary, comments and many helpful suggestions. In the revised manuscript, we have used fluorimetry for more precise ER calcium measurements (new Figure 5), clarified the high-affinity calcium binding site concern based on our previous work, and addressed possible effects of BCR-ABL translocation kinase activity upon cellular calcium signaling using the drug Imatinib (new supplements to Figure 6 and 7).

**Public Reviews:**

**Reviewer #1 (Public review):**

The researchers conducted their study using advanced techniques. They found almost no difference in calcium binding between the two proteins and observed no impact on calcium signaling, specifically store-operated calcium entry (SOCE). The study also noted an increase in ER luminal calcium-binding chaperone proteins. Surprisingly, the authors selected flow cytometry as a technique for measurements of ER luminal calcium. Considering the limitations of this approach, it would be better to use alternative approaches.

Thank you for this suggestion. We have undertaken fluorimetry-based ER calcium measurements, which are shown in a new Figure 5. These also indicate similar ER calcium levels in CRT-KO HEK293T cells, compared to those reconstituted with wild-type CRT and CRT_Del52_.

This is particularly important as previous reports, using cells from MPN patients, indicate reduced ER luminal calcium and effects on SOCE (Blood, 2020). This issue matters because earlier research with MPN patient cells reported reduced ER luminal calcium levels and altered SOCE (Blood, 2020). How do the authors explain the difference between their results and previous findings about lower ER luminal calcium and changed SOCE in MPN patient cells expressing CRTDel52?

We thank the reviewer for asking for these clarifications. We have revised the discussion to address some of these points and also clarify the findings of the referenced study (Di Buduo et al., 2020) which did not directly measure ER calcium levels. We also discuss findings from a related study with cultured megakaryocytes from patients that indicated different effects of type I vs type II mutations (Pietra et al., 2016). In the absence of engineered controls, sample-to-sample heterogeneities in primary cells make it difficult to attribute any measured differences as direct effects of CRT mutations. Different from these experiments, by using purified proteins and ITC, our studies show that the Del52 mutant has calcium-binding characteristics resembling that of the wild-type protein. Additionally, through genetic manipulations in cell lines, our studies directly address the effects of calreticulin KO and its Del52 mutation upon ER luminal and cytosolic calcium levels, and cellular SOCE signals. We did not measure significant differences in any of these parameters between the KO cells and those reconstituted with wild-type calreticulin or the Del52 mutant. As noted by the editors, these results show that Ca2+ binding by calreticulin and SOCE in a cell are not fundamentally impacted by the type I deletion mutation.

Other studies have found that unfolded protein responses are activated in MPN cells with CRTDel52 calreticulin (see Blood, 2021), and increased UPR could account for higher levels of some ER-resident calcium-binding proteins observed here.

These points are addressed in the discussion. Either protein misfolding in cells with wild-type calreticulin deficiency or the sensing of cellular calcium perturbations could induce the expression of ER calcium-binding proteins in calreticulin-deficient cells, although we favor the latter model for the reason specified in the discussion. Regardless of the precise mechanisms underlying the expression changes in calcium-binding proteins, the upregulated factors are predicted to compensate for calreticulin deficiency and contribute to the maintenance of the overall cellular calcium homeostasis.

Overall, it remains unclear how this work improves our understanding of MPN or clarifies calreticulin's role in MPN pathophysiology.

Multiple studies referenced in the manuscript have suggested links between altered calcium signaling/binding by CRT mutants and MPN pathogenesis. Our studies indicate that ER and cytosolic calcium levels and SOCE are not directly impacted by the MPN type I CALR mutation, points noted in the abstract and discussion. Thus, calcium signaling may not play a specific role in MPN CALR mutant pathology via suggested mechanisms. We are confident that readers will find these results important for better understanding the role of calreticulin type I mutations in MPN.

**Reviewer #1 (Recommendations for the authors):**

This study aimed to express, purify, and evaluate low-affinity calcium binding by a calreticulin deletion mutant (CRTdel52) that is linked to myeloproliferative neoplasms (MPN). The researchers performed cell imaging, flow cytometry, and isothermal titration calorimetry to compare calcium binding between wild-type calreticulin and CRTDel52. They assessed cytosolic calcium levels and store-operated calcium entry (SOCE) in HET293T cells (CRT knocked-out background) and in megakaryoblastic MEG-1 cells. Additionally, they examined changes in the abundance of endoplasmic reticulum (ER) resident calcium-binding proteins in cells expressing either wild-type or mutant CRT.

This study is well executed but lacks clear relevance to MPN, and it is not clear how this work advances our knowledge of calreticulin biology. No differences were found in calcium binding between wild-type calreticulin and CRTDel52, nor was SOCE impacted. They noticed, however, a compensatory increase in the abundance of some ER resident calcium-binding proteins. The lack of any significant changes in calcium behavior between wild type and CRTDel52 is not surprising based on the known amino acid sequence of calreticulin and calreticulin mutant and based on our knowledge about CRT calcium binding in general. Consequently, it is not clear how this work advances our understanding of the pathophysiology of MPN. Calcium may not play a critical role in the MPN pathology; instead, CRTDel52 secretion and receptor signaling appear more central. Further research should address how these findings relate specifically to MPN and cell biology, in general.

Our current studies demonstrate increased expression of other calcium-binding proteins in the context of heterozygous MPN type I CALR mutations (Figure 8C) or conditions resembling homozygous MPN type I CALR mutations (Figures 8D-8G). These results, together with findings of maintained ER and cytosolic calcium levels and SOCE signals (Figures 4-7 and Figure 6, supplemental Figure 2 and Figure 7, supplemental Figure 1), indicate that altered calcium binding/signaling by Del52 does not directly contribute to MPN pathology.

What is the biological or pathophysiological relevance of the CRTDel52-KDEL construct?

The KDEL sequence is important for the ER retention of CRT (Sonnichsen et al., 1994), and its addition was expected to at least partially remedy the ER retention defect of CRT_Del52_. This point is clarified in the revised results section.

The rise in ER calcium-binding proteins is noteworthy but anticipated, given likely genetic changes from UPR pathway activation in these cells. Is this relevant to MPN?

We suggest that increased expression of other calcium-binding proteins in the context of heterozygous MPN type I CALR mutations (Figure 8C) or conditions resembling homozygous MPN type I CALR mutations (Figures 8D-8G) would contribute to the maintenance of the cell’s calcium signaling capacity.

How do the authors explain the difference between their results and previous findings about lower ER luminal calcium and changed SOCE in MPN patient cells expressing CRTDel52?

The findings related to SOCE are addressed in the points discussed above and in the revised discussion. Related to ER luminal calcium, the study by Ibarra et al. (Ibarra et al., 2022) reported that CRT_Del52_ overexpressed in U2OS cells (expressing endogenous CRT) had reduced ER calcium levels compared to the same cells expressing WT CRT or CRT_Ins5_. Those measurements did not use a ratiometric ER calcium probe, and additionally it is possible that the expression of compensatory calcium-binding proteins is more muted in cells expressing endogenous wild-type CRT.

Other studies have found that unfolded protein responses are activated in MPN cells with CRTDel52 calreticulin (see Blood, 2021), and increased UPR could account for higher levels of some ER-resident calcium-binding proteins observed here.

We agree that increased UPR could account for higher levels of some ER-resident calcium-binding proteins. As noted in the revised discussion, regardless of the precise mechanisms underlying the expression changes in calcium-binding proteins, the upregulated factors are predicted to compensate for calreticulin deficiency and contribute to the maintenance of the overall cellular calcium homeostasis.

It is not clear why flow cytometry was a choice of technique for measurements of ER calcium. Pacific Blue was detected at 405 nm excitation and 452-455 nm emission, while unbound probe signals appeared in the AmCyan channel (405 nm excitation/498 nm emission). It is unnecessary to mention fluorochrome labels (like Pacific Blue or AmCyan) for channels that are not in use. Only the channels actually utilized need to be specified: The calcium-bound GEM-CEPIA1er probe's signal was collected using the Pacific Blue channel (excitation at 405 nm, emission at 452-455 nm), while the unbound probe's signal was detected using the AmCyan channel (excitation at 405 nm, emission at 498 nm). Since it's not possible to monitor emission only at a specific wavelength with a Fortessa, could this be a different channel that is being recorded?

Additionally, due to the similar spectra and potential for bleed-through between Pacific blue and Amcyan, compensation is likely necessary. Therefore, you should include single-stained control cells containing only one probe for proper reporting. Additionally, only the "GEM-CEPIA1er probe" is displayed, while the second probe is referred to solely as "unbound".

A single genetically encoded GEM-CEPIA1er probe (Suzuki et al., 2014) was used for measuring both the bound and unbound signals. The GEM-CEPIA1er probe was excited with the 405 nm violet laser. In the methods section of the revised manuscript, the GEM-CEPIA1er probe wording is included for describing both the bound and unbound signal collections. Additionally, we have undertaken new spectrofluorimetric experiments (new Figure 5), which allow for the distinct emission peaks to be recorded corresponding to the Ca^2+^-bound and Ca^2+^-unbound signals. Similar results were obtained as reported for the flow cytometry-based experiments.

Increased expression of wild-type calreticulin compared to parental cells should impact on ER calcium content and dynamics in back-transfected HEK293T-KO or MEG-1 cells. Direct ER calcium measurements in HEK293 cells with various calreticulin constructs would significantly strengthen this presentation.

Our experiments were structured to compare calcium signaling in cells expressing only wild-type CRT or CRT_Del52_ (resembling homozygous type I MPN CALR mutations) compared to CRT-KO cells. The over-expression of CRT in the reconstituted cells compared to endogenous expression level is a limitation of our study which we have acknowledged in the revised manuscript discussion. Understanding the effects of over-expression of wild-type CRT vs the CRT_Del52_ mutant upon ER and cytosolic calcium signals and SOCE is interesting, but beyond the scope of the present study.

The authors should examine the immunolocalization of CRTDel52 and wild-type protein in HEK293 cells.

Previous published studies from another lab showed that CRT_Del52_ is secreted from HEK cells and that the addition of a KDEL sequence to CRT_Del52_ reduces secretion and induces its increased intracellular accumulation (Arshad and Cresswell, 2018). This point is noted in the revised results section and the reference is cited. This appears to be the general theme in primary cells and cell lines. Previous studies and our own prior published studies have shown that CRT_Del52_ (but not wild-type CRT) is detectable in the media of cell lines and patient serum as well on the cell surface of primary cells and cell lines (Kaur et al., 2024, Venkatesan et al., 2021, Pecquet et al., 2023).

SDS-PAGE of purified proteins is overloaded, and chromatograms show extra peaks or shoulders, making protein quality assessment uncertain.

Representative chromatograms, peaks corresponding to protein monomers used for ITC analyses and the relevant gels are clarified in the revised manuscript. In new analyses since the original submission, intact protein mass spectrometry was undertaken for CRT_Del52_. The results indicate a 35-42 amino acid truncation in different preparations. The truncated proteins would still include acidic residues (between 340–351) previously implicated in low-affinity calcium binding by murine CRT that are shared between wild-type and CRT_Del52_. This new information is now included in the revised results section.

Analysis of SOCE in calreticulin-deficient cells and cells reconstituted with calreticulin or overexpressing the protein has already been reported (PMID12324449).

The indicated reference and additional related papers examining effects of CRT deficiency and overexpression on cellular calcium signaling (Arnaudeau et al., 2002, Bastianutto et al., 1995, Mery et al., 1996, Nakamura et al., 2001) are cited in the revised manuscript.

**Reviewer #2 (Public review):**

Tagoe and colleagues present a thorough analysis of the calcium (Ca2+) binding capacity of calreticulin (CRT), an endoplasmic reticulum (ER) Ca2+-buffer protein, using a mutant version (CRT del52) found in myeloproliferative neoplasms (MPNs). The authors use purified human CRT protein variants, CRT-KO cell lines, and an MPN cell line to elucidate the differing Ca2+ dynamics, both on the level of the protein and on cell-wide Ca2+-governed processes. In sum, the authors provide new insights into CRT that can be applied to both normal and malignant cell biology.

First, the authors purify CRT protein and perform isothermal titration calorimetry to quantify the Ca2+ binding capacity of CRT. They use full-length human CRT, CRT del52, and two truncations of CRT (1-339 and 1-351, the former of which should lead to the entire loss of low-affinity Ca2+ binding). While CRT del52 has previously been shown to lead to a decrease in Ca2+ binding affinity in other models, the ITC data show that this is retained in CRT del52.

Next, the authors utilize a CRT-KO cell line with subsequent addition of CRT protein variants to validate these findings with flow cytometric analysis. Cells were transfected with a ratiometric ER Ca2+ probe, and fluorescence indicates that CRT del52 is unable to restore basal ER Ca2+ levels to the same extent as CRT wild-type. To translate these findings to MPNs, the authors perform CRT-KO in a megakaryocytic cell line, where reconstitution with either CRT variant did not cause a difference in cytosolic calcium levels. The authors further test store-operated calcium entry (SOCE), an important process for maintaining ER Ca2+ levels, in these cells, and find that CRT-KO cells have lower SOCE activity, and that this can be slightly recovered with CRT addition.

Finally, the authors ask whether other effects of CRT-KO/reconstitution can affect the cellular Ca2+ signaling pathway and levels. RNASeq analysis revealed that CRT-KO leads to an increase in various chaperone protein expressions, and that reconstitution with CRT del52 is unable to reduce expression to the same extent as reconstitution with CRT wildtype.

Strengths:

The authors provide new insights into CRT that can be applied to both normal and malignant cell biology.

We thank the reviewer for the recognition that this study is important for our understanding of both normal and malignant cell biology.

Weaknesses:

(1) The authors should consider discussing the high-affinity Ca2+ binding site more in the introduction. Can they show a proof-of-concept experiment that validates that incubation of recombinant CRT reduces the function of that high-affinity Ca2+ binding site?

In a previous study (Wijeyesakere et al., 2011), we showed that at a starting calcium concentration of 0 mM and with CaCl_2_ injections to a final concentration of 70-80 mM the measured K_D_ value was 16.6 mM for calcium binding to wild type murine calreticulin, (which has ~95% sequence identity with human calreticulin), corresponding to the high-affinity site. On the other hand, at a starting calcium concentration of 50-100 mM and CaCl_2_ injections to a final concentration of 700-850 mM, the measured K_D_ value for calcium binding to wild-type murine calreticulin was 590 mM (corresponding to the low-affinity sites). We did not observe the high-affinity sites when the starting calcium concentration was 50 mM and calcium injections were at 33 mM each; similar conditions are used in the present study. These points are clarified in the revised manuscript in the results section.

(2) For Figure 2B, do you have an explanation for why the purified proteins run higher than predicted (48-52kDa) - are these proteins still tagged with pGB1?

Yes, the purified proteins shown in Figure 2B retained a GB1 tag. This point is clarified in the revised methods.

(3) The MEG-01 cell line has the BCR:ABL1 translocation, while CRT mutations are strictly found in BCR:ABL1 negative MPNs. Could these experiments be repeated in these cells treated with imatinib to decrease these effects, or see if basal MEG-01 Ca2+ levels/activity are changed with or without imatinib?

Thank you for this important point. We have assessed cytosolic calcium levels in MEG-01 cells that were treated or not treated with imatinib in new Figure 6, supplemental Figures 1 and 2 and Figure 7, supplemental Figure (1) and show that the prior results hold in imatinib-treated cells.

References

ARNAUDEAU, S., FRIEDEN, M., NAKAMURA, K., CASTELBOU, C., MICHALAK, M. & DEMAUREX, N. 2002. Calreticulin differentially modulates calcium uptake and release in the endoplasmic reticulum and mitochondria. J Biol Chem, 277, 46696-705.

ARSHAD, N. & CRESSWELL, P. 2018. Tumor-associated calreticulin variants functionally compromise the peptide loading complex and impair its recruitment of MHC-I. J Biol Chem, 293, 9555-9569.

BASTIANUTTO, C., CLEMENTI, E., CODAZZI, F., PODINI, P., DE GIORGI, F., RIZZUTO, R., MELDOLESI, J. & POZZAN, T. 1995. Overexpression of calreticulin increases the Ca2+ capacity of rapidly exchanging Ca2+ stores and reveals aspects of their lumenal microenvironment and function. J Cell Biol, 130, 847-55.

DI BUDUO, C. A., ABBONANTE, V., MARTY, C., MOCCIA, F., RUMI, E., PIETRA, D., SOPRANO, P. M., LIM, D., CATTANEO, D., IURLO, A., GIANELLI, U., BAROSI, G., ROSTI, V., PLO, I., CAZZOLA, M. & BALDUINI, A. 2020. Defective interaction of mutant calreticulin and SOCE in megakaryocytes from patients with myeloproliferative neoplasms. Blood, 135, 133-144.

IBARRA, J., ELBANNA, Y. A., KURYLOWICZ, K., CIBODDO, M., GREENBAUM, H. S., ARELLANO, N. S., RODRIGUEZ, D., EVERS, M., BOCK-HUGHES, A., LIU, C., SMITH, Q., LUTZE, J., BAUMEISTER, J., KALMER, M., OLSCHOK, K., NICHOLSON, B., SILVA, D., MAXWELL, L., DOWGIELEWICZ, J., RUMI, E., PIETRA, D., CASETTI, I. C., CATRICALA, S., KOSCHMIEDER, S., GURBUXANI, S., SCHNEIDER, R. K., OAKES, S. A. & ELF, S. E. 2022. Type I but Not Type II Calreticulin Mutations Activate the IRE1alpha/XBP1 Pathway of the Unfolded Protein Response to Drive Myeloproliferative Neoplasms. Blood Cancer Discov, 3, 298-315.

KAUR, A., VENKATESAN, A., KANDARPA, M., TALPAZ, M. & RAGHAVAN, M. 2024. Lysosomal degradation targets mutant calreticulin and the thrombopoietin receptor in myeloproliferative neoplasms. Blood Adv, 8, 3372-3387.

MERY, L., MESAELI, N., MICHALAK, M., OPAS, M., LEW, D. P. & KRAUSE, K. H. 1996. Overexpression of calreticulin increases intracellular Ca2+ storage and decreases store-operated Ca2+ influx. J Biol Chem, 271, 9332-9.

NAKAMURA, K., ZUPPINI, A., ARNAUDEAU, S., LYNCH, J., AHSAN, I., KRAUSE, R., PAPP, S., DE SMEDT, H., PARYS, J. B., MULLER-ESTERL, W., LEW, D. P., KRAUSE, K. H., DEMAUREX, N., OPAS, M. & MICHALAK, M. 2001. Functional specialization of calreticulin domains. J Cell Biol, 154, 961-72.

PECQUET, C., PAPADOPOULOS, N., BALLIGAND, T., CHACHOUA, I., TISSERAND, A., VERTENOEIL, G., NEDELEC, A., VERTOMMEN, D., ROY, A., MARTY, C., NIVARTHI, H., DEFOUR, J. P., EL-KHOURY, M., HUG, E., MAJOROS, A., XU, E., ZAGRIJTSCHUK, O., FERTIG, T. E., MARTA, D. S., GISSLINGER, H., GISSLINGER, B., SCHALLING, M., CASETTI, I., RUMI, E., PIETRA, D., CAVALLONI, C., ARCAINI, L., CAZZOLA, M., KOMATSU, N., KIHARA, Y., SUNAMI, Y., EDAHIRO, Y., ARAKI, M., LESYK, R., BUXHOFER-AUSCH, V., HEIBL, S., PASQUIER, F., HAVELANGE, V., PLO, I., VAINCHENKER, W., KRALOVICS, R. & CONSTANTINESCU, S. N. 2023. Secreted mutant calreticulins as rogue cytokines in myeloproliferative neoplasms. Blood, 141, 917-929.

PIETRA, D., RUMI, E., FERRETTI, V. V., DI BUDUO, C. A., MILANESI, C., CAVALLONI, C., SANT'ANTONIO, E., ABBONANTE, V., MOCCIA, F., CASETTI, I. C., BELLINI, M., RENNA, M. C., RONCORONI, E., FUGAZZA, E., ASTORI, C., BOVERI, E., ROSTI, V., BAROSI, G., BALDUINI, A. & CAZZOLA, M. 2016. Differential clinical effects of different mutation subtypes in CALR-mutant myeloproliferative neoplasms. Leukemia, 30, 431-8.

SONNICHSEN, B., FULLEKRUG, J., NGUYEN VAN, P., DIEKMANN, W., ROBINSON, D. G. & MIESKES, G. 1994. Retention and retrieval: both mechanisms cooperate to maintain calreticulin in the endoplasmic reticulum. J Cell Sci, 107 (Pt 10), 2705-17.

SUZUKI, J., KANEMARU, K., ISHII, K., OHKURA, M., OKUBO, Y. & IINO, M. 2014. Imaging intraorganellar Ca2+ at subcellular resolution using CEPIA. Nat Commun, 5, 4153.

VENKATESAN, A., GENG, J., KANDARPA, M., WIJEYESAKERE, S. J., BHIDE, A., TALPAZ, M., POGOZHEVA, I. D. & RAGHAVAN, M. 2021. Mechanism of mutant calreticulin-mediated activation of the thrombopoietin receptor in cancers. J Cell Biol, 220, e202009179.

WIJEYESAKERE, S. J., GAFNI, A. A. & RAGHAVAN, M. 2011. Calreticulin is a thermostable protein with distinct structural responses to different divalent cation environments. J Biol Chem, 286, 8771-85.
